# Patterns of Gene Expression in Western Corn Rootworm (*Diabrotica virgifera virgifera*) Neonates, Challenged with Cry34Ab1, Cry35Ab1 and Cry34/35Ab1, Based on Next-Generation Sequencing

**DOI:** 10.3390/toxins9040124

**Published:** 2017-03-30

**Authors:** Haichuan Wang, Seong-il Eyun, Kanika Arora, Sek Yee Tan, Premchand Gandra, Etsuko Moriyama, Chitvan Khajuria, Jessica Jurzenski, Huarong Li, Maia Donahue, Ken Narva, Blair Siegfried

**Affiliations:** 1 Department of Agronomy and Horticulture, University of Nebraska-Lincoln, Lincoln, NE 68583-0915, USA; hwang4@unl.edu (H.W.); jessica.jurzenski@fhueng.com (J.J.); 2 Center for Biotechnology, School of Biological Sciences, UNL, Lincoln, NE 68583, USA; seyun2@unl.edu (S.E.); emoriyama2@unl.edu (E.M.); 3 Dow AgroSciences, Indianapolis, IN 46268, USA; kanikaarora316@gmail.com (K.A.); STan5@dow.com (S.Y.T.); PGandra@dow.com (P.G.); HLi2@dow.com (H.L.); Mmdonahue@dow.com (M.D.); KNarva@dow.com (K.N.); 4 Monsanto, St. Louis, MO 63167, USA; chitvank@gmail.com; 5 Entomology and Nematology Department, University of Florida, Gainesville, FL 32611-0620, USA

**Keywords:** Next Generation Sequencing (NGS), *Diabrotica virgifera virgifera*, Bt challenge, differential gene expression

## Abstract

With Next Generation Sequencing technologies, high-throughput RNA sequencing (RNAseq) was conducted to examine gene expression in neonates of *Diabrotica virgifera virgifera* (LeConte) (Western Corn Rootworm, WCR) challenged with individual proteins of the binary *Bacillus thuringiensis* insecticidal proteins, Cry34Ab1 and Cry35Ab1, and the combination of Cry34/Cry35Ab1, which together are active against rootworm larvae. Integrated results of three different statistical comparisons identified 114 and 1300 differentially expressed transcripts (DETs) in the Cry34Ab1 and Cry34/35Ab1 treatment, respectively, as compared to the control. No DETs were identified in the Cry35Ab1 treatment. Putative Bt binding receptors previously identified in other insect species were not identified in DETs in this study. The majority of DETs (75% with Cry34Ab1 and 68.3% with Cry34/35Ab1 treatments) had no significant hits in the NCBI nr database. In addition, 92 DETs were shared between Cry34Ab1 and Cry34/35Ab1 treatments. Further analysis revealed that the most abundant DETs in both Cry34Ab1 and Cry34/35Ab1 treatments were associated with binding and catalytic activity. Results from this study confirmed the nature of these binary toxins against WCR larvae and provide a fundamental profile of expression pattern of genes in response to challenge of the Cry34/35Ab1 toxin, which may provide insight into potential resistance mechanisms.

## 1. Introduction

The western corn rootworm (WCR), *Diabrotica virgifera virgifera* LeConte, is an important pest of field corn, *Zea mays* L. [1,2,3] both in terms of crop losses and costs associated with management practices. Managing corn rootworm populations to minimize risk of economic loss is extremely difficult, due in part to their unique capacity to evolve resistance to a variety of management practices including chemical insecticides [4,5,6], cultural control practices such as crop rotation [7,8], and transgenic corn hybrids expressing the Cry3Bb1 toxin [9,10]. The binary toxin, Cry34/Cry35Ab1 represents two proteins that are co-expressed in transgenic corn to control WCR and are commercialized both as single event hybrids and more recently as pyramided events with either Cry3Bb1 or mCry3A. However, with resistance to Cry3 toxins documented among WCR field populations [10,11], even the pyramided events may rely exclusively on the binary Cry34/35Ab1 toxin.

It has been suggested that Cry34Ab1 and Cry35Ab1 have specific binding sites on the brush border membrane of the rootworm midgut, and Cry34Ab1 enhances the specific binding of Cry35Ab1 [12]. Cry34Ab1 has limited toxicity by itself while Cry35Ab1 alone has no toxicity, and the binary toxin is necessary to achieve mortality of rootworm larvae in diet bioassays and to achieve root protection with transgenic maize plants [13]. The specific binding receptor(s) for these two Bt toxins have yet to be identified.

Next generation sequencing provides a simple and comprehensive approach to measure changes in expression of genes in response to environmental stressors such as insecticides [14] and Bt proteins [15,16,17,18]. The goal of this study was to identify genes responsive in western corn rootworms challenged with its individual components as well as the binary toxin, Cry34/35Ab1 which has been commercialized in transgenic corn since 2006 and which still performs effectively in the field although incomplete resistance to Cry34/Cry35Ab1 was recently reported [19]. The objective of the present research is to develop an overview of WCR genes responsive to Bt Cry34/Cry35Ab1 intoxication.

## 2. Results

### 2.1. Next Generation Sequencing

The sequencing run using the Illumina HiSeq2000 yielded a total of ~1345 million paired-end raw reads. The number of reads generated for each treatment is presented in Table S1. After removal of low quality reads (Q < 20), 287 million to 383 million (~97%) high-quality reads remained (Table S2) and were used for further analysis. All raw read data are available at the NCBI Sequence Read Archive (SRA) under Accession SRP037561 [20].

### 2.2. Mapping and Differential Expression Gene Analysis

**Figure 1:**
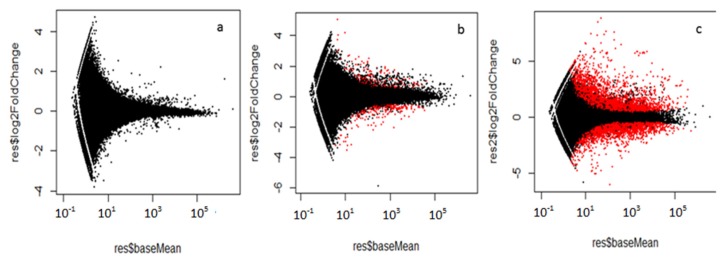
MA plots of differential expression in each treatment comparison with DESeq method. (**a**) buffer vs. Cry35Ab1; (**b**) buffer vs. Cry34Ab1; (**c**) buffer vs. Cry34/Cry35Ab1. Red dots represent the genes either up- or down-regulated at *p* (adj) < 0.05. MA plot is a way to display deferentially expressed genes versus expression strength (log2 fold change) between control and treatment.

Using the Bowtie program, nearly 70% of filtered reads for all four experimental conditions were aligned to the WCR reference transcriptome. The output (read counts) from alignment were applied directly in DESeq, and MA-plots were generated to depict a general view of the distribution of the differentially expressed transcripts (DETs) (*p* < 0.05) for all three treatments as compared to the control treatment (Figure 1). In general, transcripts with altered expression varied considerably with the source of Cry toxin challenge (Figure 1b,c). Among the three treatments, the Cry34/35Ab1 treatment produced almost 10,000 sequences defined as DETs (Figure 1c). In contrast, less than 1000 DETs were detected in the Cry34Ab1 treatment (Figure 1b) and none were detected in the treatment with Cry35 alone (Figure 1a).

The initial read count datasets were further processed by filtering transcripts with low read counts (cpm > 1) with edgeR, 26,218; 29,109 and 29,520 transcripts were remained in the Cry35Ab1, Cry34Ab1 and Cry34/35Ab1 compared to control comparisons, respectively, and further used as input in differential expression analysis.

As illustrated in Table 1, in total, 116, 132 and 135 DETs were identified with DESeq, edgeR and limma, respectively, in the Cry34Ab1treatment as compared to the control.

Among them, 114 DETs were commonly identified by these three methods. In the combined treatment with Cry34/35Ab1, 2215, 1673 and 2336 genes were classified as DETs by DESeq, edgeR and limma, respectively, and 1300 DETs were commonly identified, which is at least 10-fold greater than the number of DETs caused by exposure to Cry34Ab1 alone.

These 1300 DETs were used for subsequent analyses. As previously described, no DETs were detected by any of the three methods in the treatment with Cry35Ab1 alone.

Among the DETs identified in Cry34Ab1 (114) and Cry34/35 Ab1 (1300) treatment (Table 1), 92 DETs (31 up-regulated and 61 down-regulated) were found to be in common (Table 2). The remainder (22 and 1108 DETs) were assigned only to Cry34Ab1 or Cry34/35Ab1 treatment, respectively. Among the DETs common to both treatments, the average fold change was generally higher in the Cry34/35Ab1 treatment (Table 2).

For the unique DETs in each treatment, the average fold was similar (2.36 for Cry34Ab1 and 2.69 for Cry34/35Ab1 up-regulated genes and 3.16 and 3.3 for down-regulated genes). However, the range of fold change of DETs was greater in the Cry34/35Ab1 treatment (~6.46-fold) as compared to that in Cry34Ab treatment (3.57-fold) in down-regulated category.

#### Annotation of DETs

**Figure 2:**
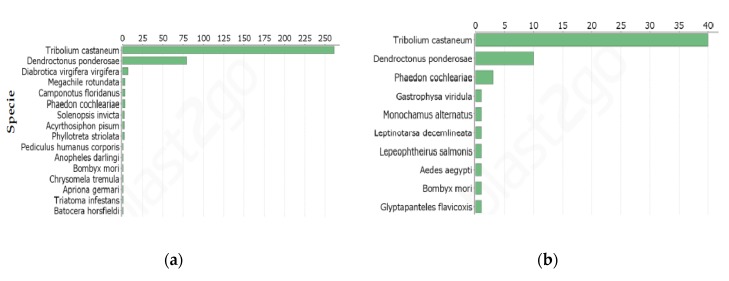
Blast hit of DGEs. (**a**) top BLAST hits of DGEs in WCR neonates exposed to Cry34/35Ab1 combination; (**b**) top BLAST hits of DGEs in WCR neonates exposed to Cry34Ab1 alone.

Nearly 70% of DETs in Cry34Ab1 treated WCR (114) and in Cry34/35Ab1 treated WCR (1300) treatment had no significant hits in the NCBI non-redundant (nr) database (Figure 2a,b). Among the DETs with hits, 35 DETs in the Cry34Ab1 treatments and 385 DETs in Cry34/35Ab1 treatments were well annotated with most hits to other Coleopterans including *Tribolium castaneum* and *Dendroctonus ponderosae*.

**Figure 3:**
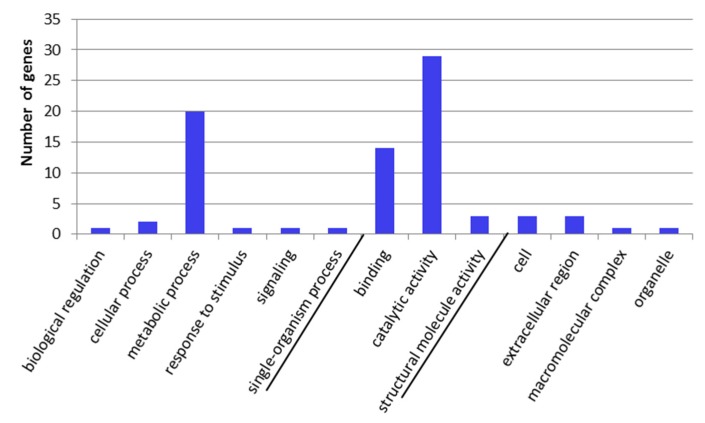
GO term categorization and distribution in Cry34Ab1 compared to control at level 2 under three main categories, i.e., biological process, molecular function and cellular component.

**Figure 4:**
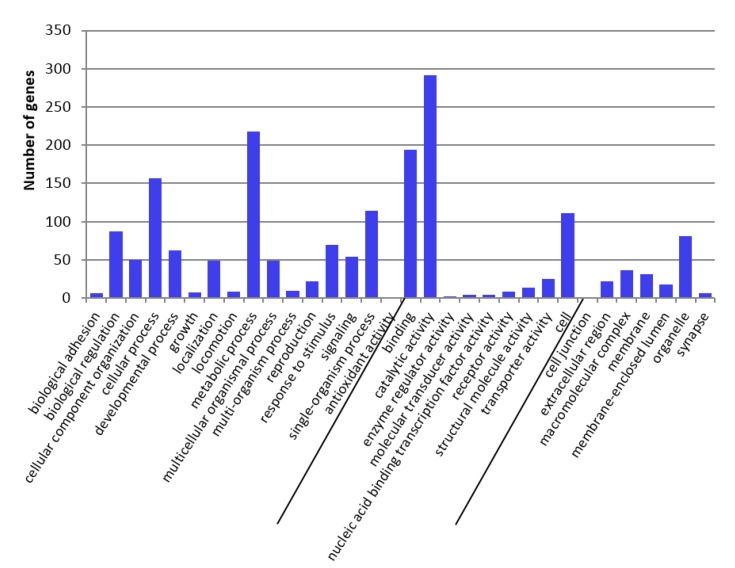
GO term categorization and distribution in Cry34/35Ab1 vs. buffer control at level 2 under three main categories, i.e. biological process, molecular function and cellular component.

Gene ontology classification of the DETs is provided in Figure 3 (Cry34Ab1 compared to control) and Figure 4 (Cry34/35Ab1 compared to control).

For the Cry34Ab1 treatment, the largest number of DETs was assigned to molecular function (17 transcripts) and cellular component (17 transcripts). Of those assigned to molecular function, catalytic activity (11 transcripts, 64.7%) and binding (6 transcripts, 35.3%) accounted for the largest number of genes assigned while all 7 transcripts represented in biological process were associated with metabolic process. In contrast, the largest group of DETs for the Cry34/35Ab1 treatment was associated with biological process (1007 transcripts), in which metabolic process (217 transcripts, 21.5%), cellular process (175 transcripts, 17.4%), and single-organism process (159 transcripts, 15.8%) accounted for the largest categories. The second largest group involved molecular function (531 transcripts), in which binding (196 transcripts, 10.7%) and catalytic activity (292 transcripts, 16%) were most abundant.

In addition to the differences in number of transcripts that responded to Cry34/35Ab1 as compared to Cry34Ab1 alone, there were 18 more additional functional activities associated with these differences in the Cry34/35Ab1 treatment (Table S3). Activities included antioxidant activity, molecular transducer activity, enzyme regulatory activity, receptor activity, transporter activity and membrane-enclosed lumen.

Transcripts coding for putative Bt toxin receptors identified from other insect species, such as cadherin, aminopeptidase N and ATP-binding cassette transporter (ABC) and metalloprotease were not detected with either Cry34Ab1 or Cry34/35Ab exposure. However, two different alkaline phosphatases (Dv_137932_c0_seq1 and Dv_149197_c0_seq1), which have been associated with Bt toxin binding in Lepidoptera, were identified as differentially expressed. The Dv_ 137932_c0_seq1 was down-regulated 7.46- and 2-fold in both Cry34/35 and Cry34Ab1 treatment, respectively. The Dv_149197_c0_seq1was up-regulated 1.65- and 1.2-fold in Cry34Ab1 and Cry34/35Ab1 treatment, respectively, based on DESeq results only.

### 2.3. GO-Term Enrichment and Pathway Analysis

As illustrated in Table S4, five significantly overrepresented GO terms were associated with DETs in Cry34Ab1 challenge. Four of these were related to molecular function, in which two GO terms were up-regulated and correlated with zinc ion binding (GO:0008270) and transition metal ion binding (GO:0046914). The other two were down-regulated and were related to hydrolase activity (GO:0004553 and GO:0016798). The remaining GO term (GO:0005975) was down-regulated and associated with carbohydrate metabolism.

In the Cry34/35Ab1 treatment, a total of 168 GO terms were significantly enriched (Tables S5 and S6). Among them, 152 GO terms (Table S5) were up-regulated and 16 (Table S6) were down-regulated. Of the up-regulated GO terms, 35 (23%) were associated with molecular function and mostly related to binding, such as ATP binding (GO:0005542), cation binding (GO:0043169), anion binding (GO:0043168), ion binding (GO:0043167) and carbohydrate derivative binding (GO:0097367). The remaining 117 GO terms were identified as biological processes and the GO terms associated with regulation accounted for the largest group (23 GO terms, 19.6%), including regulation of signaling (GO:0023051), regulation of Ras protein signal transduction (GO:0046578), regulation of hydrolase activity (GO:0051336) and regulation of lipid catabolic processes. Interestingly, only two GO terms were related to cellular process (GO:0009987) and cellular metabolic process (GO:0044237) (Table S6) were found to be under-represented as down-regulated transcripts in the Cry34/35Ab1 treatment. Moreover, no cellular component-related GO term was found to be enriched in either the Cry34Ab1 or Cry34/35Ab1 treatment.

Due to the limited annotation of the reference transcriptome, KEGG analysis was also conducted to identify pathways associated with DETs to help identify higher-level functions. For the Cry34Ab1 treatment, 7 out of 114 (6%) DETs were assigned in two pathways: (1) amino sugar and nucleotide sugar metabolism and (2) starch and sucrose metabolism. In contrast, almost 323 out of 1300 (25%) DETs associated with the Cry34/35Ab1 treatment were associated with 42 different pathways (Table S7). Among them, the majority (10 in up-regulated DETs and 22 in down-regulated DETs) of the identified were related to “metabolic pathway”. The top two pathways with most designated DETs were pyrimidine metabolism with 7 DETs and glycan degradation with 6 DETs. In addition, 6 pathways were with 5 DEGs, 9 pathways with 4 DETs, 4 pathways with 3 DETs, 9 pathways with 2 DETs assigned and the rest 28 pathways with 1 DET assigned only. In addition, among these 42 pathways identified, two pathways related to detoxification, drug metabolism-cytochrome P450 and glutathione metabolism assigned with a transcript (Dv_138610_c0_seq1) were included with a 1.61-fold change in the down-regulated category in Cry34/34Ab1 treatment.

### 2.4. Validation with RT-qPCR

For all primers used in the validation experiment for four genes, a primer efficiency value between 92.1% and 104.3% at *R*^2^ (correlation coefficient) > 0.99 was obtained (Table S8).

As shown in Table 3, the qPCR results indicated that the expression of GH45 and ALP was repressed, whereas the transcripts corresponding to the GSC and PAT were enriched in Cry34/35Ab-treated neonates. The gene expression based qPCR was in agreement with digital results from RNAseq analysis for all four genes tested.

## 3. Discussion

Differences in expression after exposure to individual toxins and the combination of the two toxins support the binary nature of the Cry34/35Ab1 toxin [21,22] as co-expression of both components in transgenic corn hybrids is necessary for control of corn rootworms. It has been recently shown that the individual toxins exhibit different binding characteristics in the midgut of rootworm and that Cry34Ab1 serves to enhance the Cry35Ab1 specific binding. Cry35Ab1 alone exhibits very low binding capacity in the absence of Cry34Ab1 [12]. Susceptible neonates challenged by exposure to individual components of the binary toxin and with an effective level of the binary toxin resulted in changes in expression that are consistent with their respective toxicities [22,23]. The combined results indicate that 1208 unique DETs were altered in their expression when challenged with the Cry34/35Ab1 combination, which was ~54 times greater than that observed in treatment with Cry34Ab1 alone (22 unique DETs). Cry35Ab1, which is non-toxic to rootworm larvae, did not cause differences in gene expression. These results are consistent with the known toxicity pattern for the two individual toxins and their combination where Cry35Ab1 is non-toxic alone, Cry34Ab1 reduces growth with much less larval mortality and Cry34/35Ab1 causes significant growth inhibition and larval mortality. The combination of the two toxins is necessary to achieve high mortality and their combined expression in transgenic maize is critical to protect roots from damage.

GO term enrichment analysis has been previously employed to identify potential pathways that respond to environmental stressors such as Bt toxins [16,18,24] and insecticides [14,25] and to classify the functions of the predicted proteins in a number of different insects [17,26]. Like other stressors, the Bt toxins induced changes in WCR gene expression [17,27,28,29]. However, the number of genes altered and GO terms enriched vary greatly with the source of challenge and target organism [16,17,24,28,30], exposure time [18], and even within different populations of the same species. For example, in two Cry1Ac resistant *Plutella xylostella* populations originating from different collections, the number of enriched GO terms associated with each population was markedly different [17]. In the current study, some of the genes responsive to Cry34/35Ab1 exposure, such as those involving zinc ion binding (GO:0008270), lipase activity (GO:0016298), catalytic activity (GO:0050790), cell communication (GO:0010646) and Ras protein signal transduction (GO:0046578), were also reported in other Bt challenge studies [16,17,18,24], suggesting expression of these genes may be common to Bt toxin exposure.

A number of putative Bt protein receptors reported among other coleopterans include cadherin-like proteins [31], ADAM metalloprotease [32] and β-glucosidase [33], and in Lepidoptera include alkaline phosphatase, cadherin, aminopeptidase N, and ABC transporters [34]. In the current study, cadherin and amino peptidase N were not detected as DETs. However, one ADAM metalloprotease (Dv_149203_c0_seq1) and β-glucosidase precursors (Dv_139888_c0_seq1) were down-regulated in both Cry34Ab1 and Cry34/35Ab1 treatment, indicating that expression was repressed.

Although not reported from other coleopterans, alkaline phosphatase has been documented as Bt receptor and associated with Bt resistance in a number of lepidopterans [35,36]. In our study, two alkaline phosphatase transcripts (Dv_149197_c0_seq1 and Dv_137932_c0_seq1) were also identified as DETs in both Cry34Ab1 and Cry34/35Ab1 treatments. The Dv_137932_c0_seq1 was down-regulated almost 3-fold (*p* < 0.05), suggesting that this alkaline phosphatase (Dv_137932_c0_seq1) is associated with toxin response.

The over expression of glutathione S-transferase (GST) has been associated with insecticide resistance in many insect pests [37]. Recent studies have also shown that the GST expression was reduced in Cry3Aa intoxicated *T. molitor* larvae [24] and Cry1Ab resistant Asian corn borer (*Ostrinia furnacalis*) [29]. In our study, one transcript, Dv_138610_c0_seq1 assigned as glutathione transferase was also down-regulated 1.6-fold (*p* < 0.05) in the Cry34/35Ab1 treatment. Among the four metabolism pathways identified in the Cry34Ab1 treatment, three of them (purine metabolism, starch and sucrose metabolism and amino sugar and nucleotide sugar metabolism) were common with pathways identified in the Cry34/35Ab1 treatment although 55 more metabolism transcripts were triggered in neonates challenged by Cry34/35Ab1 treatment. The higher number of DETs in the Cry34/35Ab1 may be indicative of higher levels of stress levels imposed by the toxic combination of the two toxins. These results are in agreement with findings by Li et al. [12], in which Cry34Ab1 enhances the binding of Cry35Ab1.

Several other pathways identified in the Cry34/35Ab1 treatment, such as nicotinate and nicotinamide metabolism, tryptophan metabolism, pyruvate metabolism and starch and sucrose metabolism, have been associated with environmental [38] and insecticide induced stress [15] in insects, indicating these pathways might be common response in insects to stressors.

## 4. Conclusions

NGS provides an important tool to investigate changes in gene expression associated with environmental challenges [24,39,40], and the combination of different statistical methods in downstream analysis of expression effects (DESeq, edgeR and limma) improved the different gene-calling results. Multiple transcripts were detected as responsive to the challenges of Cry34Ab1 and Cry34/35Ab1 combined. However, Cry35Ab1 alone did not produce a response, which is consistent with the lack of toxicity for this toxin. No previously identified Bt receptor genes (except for an alkaline phosphatase) were identified as differentially expressed, suggesting the receptor for Cry34Ab1 and Cry35Ab1 might be unique or that expression of the specific receptor is unaffected by toxin exposure.

It is also possible that actual toxin receptor(s) are not responsive to toxin exposure and that the observed differences in expression are a function of cellular stress and subsequent repair processes. Further analysis is needed to assess not only the function of those genes significantly affected by exposure to Cry34/35Ab1, but also explore the relative changes of associated proteins especially given the large percentage of DEGs that had no annotation. The data obtained herein should facilitate a better understanding of the active response to Cry toxin challenge at a transcriptomic level and provide new insights into the interaction of WCR and the Cry34/35Ab1 binary toxin.

## 5. Materials and Methods

### 5.1. Insects

The susceptible western corn rootworm eggs from a non-diapause WCR strain, which has been reared continuously for more than 30 years in the absence of insecticide and any Bt toxins exposure, were purchased from Crop Characteristics, Inc. (Farmington, MN, USA) and incubated in a growth chamber at 26 ± 1 °C and 60% ± 10% relative humidity with a photoperiod of 12:12 h (L:D) until hatching occurred approximately two weeks later.

### 5.2. Bt Proteins

Full-length Cry35Ab1 protein (44 kDa) was digested with chymotrypsin to generate active protein core fragments. Briefly, full-length Cry35Ab1 was incubated with bovine pancreatic chymotrypsin (Sigma, St. Louis, MO, USA) at 50:1 (*w*/*w* ratio = Cry protein:enzyme) in 100 mM sodium citrate buffer, pH 3.0, at 4 °C with gentle shaking for 2–3 days. The resulting core fragment (40 kDa) was analyzed on a 12% SDS-PAGE gel as described by Crespo et al. [41]. The activated Cry35Ab1 and full-length Cry34Ab1were used for all experiments in this study.

### 5.3. Exposure

Exposure experiments were conducted in 24 well cell culture plates (Costa 3526, Corning Incorporated, NY, USA). One mL of Dow AgroSciences proprietary corn rootworm diet was dispensed into wells of each plate and the surface was coated with Bt protein(s) at 15 µg/cm^2^ for Cry34Ab1 or Cry35Ab1 alone and 15 µg/cm^2^ Cry34Ab1 + 15 µg/cm^2^ Cry35Ab1 combined. The Cry toxins were diluted in 20 mM sodium acetate solution at pH 3.5. Controls consisted of wells treated with sodium acetate solution only. Six replicates for each treatment, including controls, were prepared for a total of 24 samples. Approximately 32 neonates (<24 h after hatching) were transferred into each pre-coated well with a fine camel hair paint brush and were exposed to Bt protein(s) for 48 h at room temperature. All living neonates in each well were pooled, snap frozen in liquid nitrogen and stored at −80 °C until RNA extraction.

### 5.4. RNA Isolation

Total RNA was extracted from pooled samples using the RNeasy Mini Kit (Cat. 74104, Qiagen, Germantown, MD, USA) and treated with RNase-Free Dnase (Cat. 79254, Qiagen) to eliminate DNA contamination according to the manufacturer’s instructions. The quality of RNA samples was evaluated on 1% agrose gels and quantity was estimated on NanoDrop-1000 (Thermo Fisher Scientific, Bartlesville, OK, USA) before submission for RNAseq analysis.

### 5.5. Next Generation Sequencing

The RNA sample integrity of all twenty-four RNA samples was further assessed using an Agilent 2100 Bioanalyzer (Cat. G2940CA, Agilent Technologies, Richardson, TX, USA) at the Next Generation Sequencing Core Facility at Durham Research Center, University of Nebraska Medical Center and were processed for library construction and paired end sequencing on an Illumina HiSeq2000 system (San Diego, CA, USA). All samples were sequenced using 100 bp paired-end reads.

### 5.6. Read Mapping and Differential Expression Analysis

A stringent quality filter process was applied by removing reads that did not have a minimum Phred quality score (Q64) of 20 per base corresponding to a 1% expected error rate using Sickle/1.2 (version 1.2, San Francisco, USA, [42]) according to the manual instructions. To map the quality reads back to WCR reference transcriptome contigs [20], the Bowtie aligner (2013, version 1.0.0, Baltimore, MD, USA, [43]), Samtools (version 1.3, La Jolla, CA, USA, [44]) was used to retrieve read counts for all treatments and control. All data analyses were performed at Holland Computing Center (HCC) at the University of Nebraska.

To identify differentially expressed genes among the treatments, three commonly used statistical methods (DESeq [45], edgeR [46] and limma [47]) for detecting differential expression in RNA-seq studies were employed [48]. For each treatment, only differentially expressed transcripts (DETs) that were identified by all three methods were used in further analysis. The edgeR package was initially used to remove low read counts from all 24 samples at a threshold of >1 cpm (count per million). All filtered data were used as input for differential expression analysis at adjusted *p* < 0.05 (hereafter *p* < 0.05). For edgeR and limma, TMM normalization was used to identify differentially expressed genes. For DESeq, the size factor normalization was used.

### 5.7. Homology Searches and Gene Ontology Analysis

The resulting DETs from differential expression analysis described above were annotated using the BLASTx algorithm against the NCBI nonredundant (nr) database with a stringent *e*-value threshold cut-off of 10^−25^. For gene ontology analysis, BLAST2GO [49] was employed for further analysis at default settings. With the annotated WCR transcriptome database as reference, the GO enrichment analyses were conducted using the Fisher’s Exact Test implemented in the functional enrichment feature of BLAST2GO at default settings (FDR < 0.05) [49].

### 5.8. Validation of Gene Expression via qPCR

Two up-regulated DEGs, GSC (gut-specific chitinase, 12.55-fold) and PAT (proton-coupled amino acid transporter 1-like, 8.69-fold) and two down-regulated DEGs, GH45 (endo-beta glucansase 32-fold change) and ALP (alkaline phosphatase, 7.4-fold) identified in both Cry34Ab1 and Cry34/35Ab1 treatments were selected for validation by qRT-PCR analysis. The qPCR primers for target genes were designed with a web-based tool (Primer3plus, 2012, Singapore, Singapore [50]). The actin gene was used as endogenouse gene (housekeeping gene) and the primer efficiency tests were conducted as described by Rodrigues et al. [51]. Primers with efficiency between 90 and 110% were selected for qPCR (Table S8). The cDNA used in this validation experiment were synthesized with a different batch of RNA prepared from different samples collected in Exposure step previously. The RNAs from three biological samples from Cry34/35Ab1 treatment and control (buffer) were used for cDNA synthesis with QuantiTect Rev Transcription Kit (Qiagen, Cat. 205311) according to the manufacture’s instruction. The 2^−ΔΔC^_T_ method was used to calculate the relative expression of target genes [52].

## Figures and Tables

**Table 1:** RNAseq differential gene expression for WCR exposed to Cry34/35Ab1 toxin.

Analysis Method	Cry34Ab1 vs. Buffer				Cry35Ab1 vs. Buffer			Cry34Ab1 + Cry35Ab1 vs. Buffer			
	up	down	total	Shared *	up	down	total	up	down	total	shared
**DESeq**	44	72	116	114	0	0	0	992	1223	2215	1300
**edgeR**	49	83	132		0	0	0	647	1026	1673	
**limma**	48	87	135		0	0	0	1093	1243	2336	

* indicate the total DETs shared among three analysis method.

**Table 2:** Number of DETs shared and not shared between Cry34Ab and Cry34Ab1 + Cry35Ab1 treatment.

Category	Category	# Contigs		Digital Expression in Fold Change *	
up-regulated	shared	31		2.55 (2–4.66) ^a^	4.5 (2.3–12.5) ^b^
	unique	12 ^a^	534 ^b^	2.36 (2.14–2.9) ^a^	2.69 (2–64) ^b^
down-regulated	Shared	61		2.64 (2–5.6) ^a^	10.6 (2.49–194) ^b^
	unique	10 ^a^	674 ^b^	3.16 (2–1) ^a^	3.3 (2–88) ^b^

^a^ challenged with Cry34Ab; ^b^ challenged with combination of Cry34/35Ab1; * all fold changes are from DESeq data at *p* < 0.05).

**Table 3:** Comparison of expression of four randomly selected genes with two analysis methods.

Category	Gene	Fold in RNAseq Analysis	Fold Change in qPCR
up-regulated	GSC	12.55 *	91.01 *
	PAT	8.69 *	3.44 *
down-regulated	GH45	32 *	0.31 *
	ALP	7.4 *	0.16 *

* The fold change either in RNAseq analysis or qPCR was from Cry34/35-treated samples and was compared to the expression of the same gene from the control; Abbreviation: GSC-gut-specific chitinase, PAT-proton-coupled amino acid transporter 1-like, GH45-endo-beta glucansase and ALP-alkaline phosphatase.

## Supplementary Materials

The following are available online at www.mdpi.com/2072-6651/9/4/124/s1, Table S1: Reads generated on RNAseq for each treatment and control, Table S2: Summary of illumina generated read production and read for mapping after filtering, Table S3: 18 unique functional activities associated with 34/35Ab1 treatment as compared to 34Ab1 treatment, Table S4: Summary of GO term enrichment results of significantly regulated genes in treatment with Cry34Ab1 at FDR < 0.05, Table S5: Summary of GO term enrichment results of significantly up-regulated DEGs in treatment with Cry34/35Ab1 at FDR < 0.05, Table S6: Summary of GO term enrichment results of significantly down-regulated DEGs in treatment with Cry34/35Ab1 at FDR < 0.05, Table S7: KEGG pathways involving 323 DEGs in Cry34/35Ab1 treatment as compared to the control, Table S8: Primers used in validation of gene expression via qPCR.
