# Supplementary material for: Patterns of Gene Expression in Western Corn Rootworm (Diabrotica virgifera virgifera) Neonates, Challenged with Cry34Ab1, Cry35Ab1 and Cry34/35Ab1, Based on Next-Generation Sequencing
Source: Toxins (Basel). 2017 Mar 30;9(4):124. doi: 10.3390/toxins9040124 (PMC5408198; doi:10.3390/toxins9040124)
Supplement: Supplementary file 1 [file toxins-09-00124-s001.pdf]

Supplementary Materials: Patterns of Gene Expression in Western Corn Rootworm (*Diabrotica virgifera virgifera*) Neonates, Challenged with Cry34Ab1, Cry35Ab1 and Cry34/35Ab1, Based on Next-Generation Sequencing

Haichuan Wang, Seong-il Eyun, Kanika Arora, Sek Yee Tan, Premchand Gandra, Etsuko Moriyama, Chitvan Khajuria, Jessica Jurzenski, Huarong Li, Maia Donahue, Ken Narva and Blair Siegfried

Table S1. Reads generated on RNAseq for each treatment and control.

Treatment	Replicates	Raw reads	Reads after filtering	Treatment	Replicates	Raw reads	Reads after filtering
**34Ab1**	1	43,878,467	43,000,697	**35Ab1**	1	52,627,083	51,337,801
	2	67,453,316	66,138,309		2	72,377,483	71,022,040
	3	54,090,779	52,645,383		3	60,588,973	59,204,754
	4	46,499,131	45,536,519		4	95,534,011	93,551,718
	5	58,632,657	57,607,911		5	55,244,454	54,360,811
	6	60,566,736	59,306,183		6	54,383,911	53,574,954
**34/35Ab1**	1	49,958,689	48,322,143	**Control**	1	43,886,263	42,965,828
	2	73,623,445	72,094,127		2	52,412,797	51,105,386
	3	68,986,765	67,703,519		3	55,045,308	53,941,838
	4	39,331,805	38,387,901		4	42,630,516	41,576,918
	5	34,628,586	33,655,979		5	54,292,321	53,020,759
	6	64,366,787	63,342,959		6	46,251,448	45,015,806

Table S2. Summary of Illumina generated read production and read for mapping after filtering.

	**34**	**35**	**34/35 Control**	**34**
No. of reads	331 million	390 million	330 million	294 million
read length (bp)	101	101	101	101
No. of reads after filtering	324 million	383 million	323 million	287 million
percentage retained	97.9	98	97.7	97.6

Table S3. 18 unique functional activities associated with 34/35Ab1 treatment as compared to 34Ab1 treatment.

**antioxidant activity**
biological adhesion
cell junction
cellular component organization or biogenesis
developmental process
enzyme regulator activity growth
localization
membrane
membrane-enclosed lumen
molecular transducer activity
multicellular organismal process
multi-organism process
nucleic acid binding transcription factor activity
receptor activity
reproduction
synapse
transporter activity

Table S4. Summary of GO term enrichment results of significantly regulated genes in treatment with Cry34Ab1 at FDR < 0.05.

**In Up-regulated Category**				
GO-ID	Term	Category	FDR	Over/Under [a]
GO:0008270	zinc ion binding	F	2.19E-02	over
GO:0046914	transition metal ion binding	F	2.33E-02	over
**In Down-regulated Category**				
GO-ID	Term	Category	FDR	Over/Under [a]
GO:0004553	hydrolase activity	F	1.43E-02	over
GO:0016798	hydrolase activity	F	2.00E-02	over
GO:0005975	carbohydrate metabolic process	P	0.00245	over

[a] over/under indicates if this GO term is enriched or not as compared to WCR transcriptome reference.

Table S5. Summary of GO term enrichment results of significantly up-regulated DEGs in treatment with Cry34/35Ab1 at FDR < 0.05.

**GO-ID**	**Term**	**Category**	**FDR**	**Over/Under [a]**
GO:0003878	ATP citrate synthase activity	F	2.33E-06	OVER
GO:0004775	succinate-CoA ligase (ADP-forming) activity	F	2.33E-06	OVER
GO:0004774	succinate-CoA ligase activity	F	2.33E-06	OVER
GO:0016405	CoA-ligase activity	F	3.26E-06	OVER
GO:0016878	acid-thiol ligase activity	F	3.26E-06	OVER
GO:0046912	transferase activity	F	9.73E-06	OVER
GO:0016877	ligase activity, forming carbon-sulfur bonds	F	1.9E-05	OVER
GO:0043167	ion binding	F	4.29E-05	OVER
GO:0005524	ATP binding	F	0.000167	OVER
GO:0032559	adenyl ribonucleotide binding	F	0.000167	OVER
GO:0030554	adenyl nucleotide binding	F	0.000207	OVER
GO:0035639	purine ribonucleoside triphosphate binding	F	0.000342	OVER
GO:0032550	purine ribonucleoside binding	F	0.000342	OVER

GO:0032555	purine ribonucleotide binding	F	0.000342	OVER
GO:0001883	purine nucleoside binding	F	0.000342	OVER
GO:0032549	ribonucleoside binding	F	0.000342	OVER
GO:0001882	nucleoside binding	F	0.000342	OVER
GO:0032553	ribonucleotide binding	F	0.000351	OVER
GO:0017076	purine nucleotide binding	F	0.000374	OVER
GO:0097367	carbohydrate derivative binding	F	0.000443	OVER
GO:0043168	anion binding	F	0.001521	OVER
GO:0004767	sphingomyelin phosphodiesterase activity	F	0.001927	OVER
GO:0004672	protein kinase activity	F	0.002168	OVER
GO:0005388	calcium-transporting ATPase activity	F	0.00369	OVER
GO:0016874	ligase activity	F	0.006764	OVER
GO:0016740	transferase activity	F	0.008216	OVER
GO:0004620	phospholipase activity	F	0.008521	OVER
GO:0016773	phosphotransferase activity	F	0.009478	OVER
GO:0016298	lipase activity	F	0.013152	OVER
GO:0015662	ATPase activity	F	0.02097	OVER
GO:1901265	nucleoside phosphate binding	F	0.022184	OVER
GO:0000166	nucleotide binding	F	0.022184	OVER
GO:0036094	small molecule binding	F	0.024149	OVER
GO:0016301	kinase activity	F	0.027656	OVER
GO:0043169	cation binding	F	0.03705	OVER
GO:0016477	cell migration	P	1.23E-05	OVER
GO:0009395	phospholipid catabolic process	P	4.29E-05	OVER
GO:0006685	sphingomyelin catabolic process	P	4.29E-05	OVER
GO:0006684	sphingomyelin metabolic process	P	4.29E-05	OVER
GO:0006665	sphingolipid metabolic process	P	4.29E-05	OVER
GO:0030149	sphingolipid catabolic process	P	4.29E-05	OVER
GO:0046466	membrane lipid catabolic process	P	4.29E-05	OVER
GO:0007015	actin filament organization	P	4.67E-05	OVER
GO:0046578	regulation of Ras protein signal transduction	P	5.82E-05	OVER
GO:0007265	Ras protein signal transduction	P	7.15E-05	OVER
GO:0051674	localization of cell	P	7.77E-05	OVER
GO:0048870	cell motility	P	7.77E-05	OVER
GO:0040011	locomotion	P	0.000117	OVER
GO:0030036	actin cytoskeleton organization	P	0.00025	OVER
GO:0030029	actin filament-based process	P	0.000281	OVER
GO:0051056	regulation of small GTPase mediated signal transduction	P	0.00029	OVER
GO:0046434	organophosphate catabolic process	P	0.000332	OVER
GO:0045454	cell redox homeostasis	P	0.000343	OVER
GO:1901565	organonitrogen compound catabolic process	P	0.000381	OVER
GO:1902531	regulation of intracellular signal transduction	P	0.000443	OVER
GO:0042439	ethanolamine-containing compound metabolic process	P	0.000443	OVER
GO:1901616	organic hydroxy compound catabolic process	P	0.000443	OVER

GO:0046164	alcohol catabolic process	P	0.000443	OVER
GO:0007155	cell adhesion	P	0.000459	OVER
GO:0022610	biological adhesion	P	0.000459	OVER
GO:0044712	single-organism catabolic process	P	0.001019	OVER
GO:0043087	regulation of GTPase activity	P	0.001019	OVER
GO:0033124	regulation of GTP catabolic process	P	0.001019	OVER
GO:0030811	regulation of nucleotide catabolic process	P	0.001019	OVER
GO:0009118	regulation of nucleoside metabolic process	P	0.001019	OVER
GO:0033121	regulation of purine nucleotide catabolic process	P	0.001019	OVER
GO:1900542	regulation of purine nucleotide metabolic process	P	0.001043	OVER
GO:0006140	regulation of nucleotide metabolic process	P	0.001045	OVER
GO:0031329	regulation of cellular catabolic process	P	0.001163	OVER
GO:0009894	regulation of catabolic process	P	0.00124	OVER
GO:0009966	regulation of signal transduction	P	0.00152	OVER
GO:0044242	cellular lipid catabolic process	P	0.00152	OVER
GO:0044248	cellular catabolic process	P	0.00152	OVER
GO:0010646	regulation of cell communication	P	0.001521	OVER
GO:0023051	regulation of signaling	P	0.001521	OVER
GO:0006643	membrane lipid metabolic process	P	0.001713	OVER
GO:0048583	regulation of response to stimulus	P	0.001927	OVER
GO:0006468	protein phosphorylation	P	0.001927	OVER
GO:0007010	cytoskeleton organization	P	0.001927	OVER
GO:0043603	cellular amide metabolic process	P	0.002602	OVER
GO:0006576	cellular biogenic amine metabolic process	P	0.00296	OVER
GO:0019637	organophosphate metabolic process	P	0.00296	OVER
GO:0019220	regulation of phosphate metabolic process	P	0.003017	OVER
GO:0051174	regulation of phosphorus metabolic process	P	0.003017	OVER
GO:1901575	organic substance catabolic process	P	0.003058	OVER
GO:0009056	catabolic process	P	0.003708	OVER
GO:0019725	cellular homeostasis	P	0.004193	OVER
GO:0051336	regulation of hydrolase activity	P	0.004193	OVER
GO:0009308	amine metabolic process	P	0.004469	OVER
GO:0044106	cellular amine metabolic process	P	0.004469	OVER
GO:0016042	lipid catabolic process	P	0.004978	OVER
GO:0071822	protein complex subunit organization	P	0.00499	OVER
GO:0007264	small GTPase mediated signal transduction	P	0.005543	OVER
GO:0006928	cellular component movement	P	0.006837	OVER
GO:0042592	homeostatic process	P	0.007285	OVER
GO:0044262	cellular carbohydrate metabolic process	P	0.00827	OVER
GO:1901564	organonitrogen compound metabolic process	P	0.009478	OVER
GO:1901069	guanosine-containing compound catabolic process	P	0.010194	OVER
GO:0006184	GTP catabolic process	P	0.010194	OVER
GO:0009261	ribonucleotide catabolic process	P	0.01085	OVER

GO:0009207	purine ribonucleoside triphosphate catabolic process	P	0.01085	OVER
GO:0009203	ribonucleoside triphosphate catabolic process	P	0.01085	OVER
GO:0009154	purine ribonucleotide catabolic process	P	0.01085	OVER
GO:0009146	purine nucleoside triphosphate catabolic process	P	0.01085	OVER
GO:0006195	purine nucleotide catabolic process	P	0.01085	OVER
GO:0006152	purine nucleoside catabolic process	P	0.01085	OVER
GO:0046130	purine ribonucleoside catabolic process	P	0.01085	OVER
GO:0009143	nucleoside triphosphate catabolic process	P	0.01085	OVER
GO:0042454	ribonucleoside catabolic process	P	0.01085	OVER
GO:0009164	nucleoside catabolic process	P	0.01085	OVER
GO:1901658	glycosyl compound catabolic process	P	0.01085	OVER
GO:0072523	purine-containing compound catabolic process	P	0.01085	OVER
GO:0005975	carbohydrate metabolic process	P	0.010887	OVER
GO:0046039	GTP metabolic process	P	0.010952	OVER
GO:0009166	nucleotide catabolic process	P	0.011213	OVER
GO:1901292	nucleoside phosphate catabolic process	P	0.011213	OVER
GO:1901068	guanosine-containing compound metabolic process	P	0.011213	OVER
GO:1901136	carbohydrate derivative catabolic process	P	0.013152	OVER
GO:0009205	purine ribonucleoside triphosphate metabolic process	P	0.014041	OVER
GO:0009144	purine nucleoside triphosphate metabolic process	P	0.014041	OVER
GO:0034655	nucleobase-containing compound catabolic process	P	0.014041	OVER
GO:0009199	ribonucleoside triphosphate metabolic process	P	0.014383	OVER
GO:0044270	cellular nitrogen compound catabolic process	P	0.014383	OVER
GO:0046700	heterocycle catabolic process	P	0.014495	OVER
GO:0019439	aromatic compound catabolic process	P	0.014495	OVER
GO:1901361	organic cyclic compound catabolic process	P	0.014732	OVER
GO:0009141	nucleoside triphosphate metabolic process	P	0.014972	OVER
GO:1902589	single-organism organelle organization	P	0.015844	OVER
GO:0046128	purine ribonucleoside metabolic process	P	0.015971	OVER
GO:0042278	purine nucleoside metabolic process	P	0.016099	OVER
GO:0009119	ribonucleoside metabolic process	P	0.017853	OVER
GO:0044281	small molecule metabolic process	P	0.019454	OVER
GO:0050790	regulation of catalytic activity	P	0.020791	OVER
GO:0009150	purine ribonucleotide metabolic process	P	0.021374	OVER
GO:0009116	nucleoside metabolic process	P	0.021374	OVER
GO:1901657	glycosyl compound metabolic process	P	0.021374	OVER
GO:0065009	regulation of molecular function	P	0.021374	OVER
GO:0006066	alcohol metabolic process	P	0.021423	OVER

GO:0006163	purine nucleotide metabolic process	P	0.021501	OVER
GO:0044763	single-organism cellular process	P	0.021598	OVER
GO:0009259	ribonucleotide metabolic process	P	0.022184	OVER
GO:0019693	ribose phosphate metabolic process	P	0.022184	OVER
GO:0035556	intracellular signal transduction	P	0.022596	OVER
GO:0072521	purine-containing compound metabolic process	P	0.023217	OVER
GO:1901615	organic hydroxy compound metabolic process	P	0.027656	OVER
GO:0044282	small molecule catabolic process	P	0.028892	OVER
GO:0043933	macromolecular complex subunit organization	P	0.029575	OVER
GO:0009117	nucleotide metabolic process	P	0.040393	OVER
GO:0006753	nucleoside phosphate metabolic process	P	0.040666	OVER
GO:0016310	phosphorylation	P	0.042679	OVER
GO:0050794	regulation of cellular process	P	0.047028	OVER
GO:0055086	nucleobase-containing small molecule metabolic process	P	0.049905	OVER

[a] over/under indicates if this GO term is enriched or not as compared to WCR transcriptome reference.

Table S6. Summary of GO term enrichment results of significantly down-regulated DEGs in treatment with Cry34/35Ab1 respectively at FDR < 0.05.

GO-ID	Term	Category	FDR	Over/Under [a]
GO:0016798*	hydrolase activity, acting on glycosyl bonds	F	1.24E-07	OVER
GO:0004563	beta-N-acetylhexosaminidase activity	F	7.48E-06	OVER
GO:0004553*	hydrolase activity, hydrolyzing O-glycosyl compounds	F	7.48E-06	OVER
GO:0015929	hexosaminidase activity	F	8.48E-06	OVER
GO:0016787	hydrolase activity	F	0.017657	OVER
GO:0004523	RNA-DNA hybrid ribonuclease activity	F	0.017745	OVER
GO:0006032	chitin catabolic process	P	7.48E-06	OVER
GO:0006026	aminoglycan catabolic process	P	8.48E-06	OVER
GO:1901072	glucosamine-containing compound catabolic process	P	1.04E-05	OVER
GO:0046348	amino sugar catabolic process	P	1.04E-05	OVER
GO:0005975*	carbohydrate metabolic process	P	0.00245	OVER
GO:0006030	chitin metabolic process	P	0.004376	OVER
GO:0006022	aminoglycan metabolic process	P	0.005214	OVER
GO:1901071	glucosamine-containing compound metabolic process	P	0.005214	OVER
GO:0006040	amino sugar metabolic process	P	0.005214	OVER
GO:0009057	macromolecule catabolic process	P	0.03855	OVER
GO:0009987	cellular process	P	0.000419	UNDER
GO:0044237	cellular metabolic process	P	0.020641	UNDER

[a] over/under indicates if this GO term is enriched or not as compared to WCR transcriptome reference.
* the GO terms highlighted in light brown were shared between Cry34Ab1 and Cry34/35Ab1 treatment.

Table S7. KEGG pathways involving 323 DEGs in Cry34/35Ab1 treatment as compared to control.

Pathways	#Seqs	Category
Carbon fixation pathways in prokaryotes	5	up-regulated
Pyrimidine metabolism	5	
Citrate cycle (TCA cycle)	5	
Propanoate metabolism	4	
Purine metabolism	4	
Nicotinate and nicotinamide metabolism	4	
C5-Branched dibasic acid metabolism	4	
Sphingolipid metabolism	3	
Tyrosine metabolism	2	
Isoquinoline alkaloid biosynthesis	2	
Oxidative phosphorylation	1	
Glyoxylate and dicarboxylate metabolism	1	
Clycine, serine and threonine metabolism	1	
T cell receptor signaling pathway	1	
Porphyrin and chlorophyll pathway	1	
Glutathione metabolism	1	
Tryptophan metabolism	1	
Pyrimidine metabolism	7	down-regulated
Other glycan degradation	6	
Starch and sucrose metabolism	5	
One carbon pool by folate	5	
Amino sugar and nucleotide sugar metabolism	5	
Various types of N-glycan biosynthesis	4	
Glycosphingolipid biosynthesis-ganglio	4	
Glycosphingolipid biosynthesis-globo serine	4	
Glycosaminoglycan degradation	4	
Purine metabolism	4	
Glutathione metabolism	3	
Clycine, serine and threonine metabolism	3	
Phenylalanine metabolism	3	
Glycolysis/Gluconeogenesis	2	
Phenylpropanoid biosynthesis	2	
Histidine metabolism	2	
Thiamine metabolism	2	
Fatty acid degradation	2	
Tryptophan metabolism	2	
Lysine degradation	2	down-regulated
Carbon fixation in photosynthetic organisms	1	
Inositol phosphate metabolism	1	
Glycerolipid metabolism	1	
Arginine metabolism	1	
Propanoate metabolism	1	

Isoquinoline alkaloid biosynthesis	1
Betalain biosynthesis	1
Pentose and glucuronate interconversions	1
Cysteine and methionine metabolism	1
Ascorbate and aldarate metabolism	1
Tyrosine metabolism	1
Fructose and mannose metabolism	1
Limonene and pinene degradation	1
Valine, leucine and isoleucine degradation	1
Indole alkaloid biosynthesis	1
Terpenoid backbone biosynthesis	1
Arachidonic acid metabolism	1
Drug metabolism-cytochrome P450	1
Metabolism of xenobiotics by cytochrome P450	1
Chloroalkane and chloroalkene degradation	1
Pyruvate metabolism	1

Table S8. Primers used in validation of gene expression via qPCR.

Primer	oligoes (5`-3`)	Primer efficiency
Dv_GH45_qPCR_F	GGGTATGTTGCTGCTCGTTT	96%
Dv_GH45_qPCR_R	TTGTTTCAGGTGCATTTCCA	
Dv_ALP_qPCR_F	ACGAAATCGCTGCAGAAAAG	104.3%
Dv_ALP_qPCR_R	TGGACATTCCATCTCCAAGA	
Dv_AAtransporter_qPCR_F	CTACGCAGGTGGACTCACAA	92.1%
Dv_AAtransporter_qPCR_R	GGTCGGTGTATTCGTCACCT	
Dv_gutChitinase_qPCR_F	AGCTGCAAATGCAATGTTGA	102%
Dv_gutChitinase_qPCR_R	AAAATCGTCCTTGTCGATGG	
